# Predictive value of the systemic inflammation grade for overall survival in patients with colorectal cancer after surgery: outperforming NLR and mGPS

**DOI:** 10.3389/fonc.2025.1529670

**Published:** 2025-06-17

**Authors:** Jianing Wang, Yujun Liu, Wenliang Jiang, Dongli Zhang, Chao Cheng, Cuixia Liu, Zhibin Zhao, Honggang Wang

**Affiliations:** ^1^ Department of General Surgery, Beidahuang Industry Group General Hospital, Harbin, Heilongjiang, China; ^2^ Department of General Surgery, The Affiliated Taizhou People’s Hospital of Nanjing Medical University, Taizhou School of Clinical Medicine, Nanjing Medical University, Taizhou, Jiangsu, China; ^3^ Department of Gastroenterology, The Affiliated Taizhou People’s Hospital of Nanjing Medical University, Taizhou School of Clinical Medicine, Nanjing Medical University, Taizhou, Jiangsu, China

**Keywords:** systemic inflammation grade, colorectal cancer, prognosis, neutrophil-to-lymphocyte ratio, modified Glasgow prognostic score, overall survival

## Abstract

**Background:**

Accurate prognostic stratification remains challenging in colorectal cancer (CRC) patients after curative resection. The Systemic Inflammation Grade (SIG), integrating neutrophil-to-lymphocyte ratio (NLR) and modified Glasgow Prognostic Score (mGPS), was proposed as a composite marker to refine risk assessment.

**Methods:**

This retrospective study analyzed 263 CRC patients undergoing R0 resection (2015–2019). Preoperative NLR and mGPS were calculated, and SIG was categorized into low (0), medium (1), and high (≥2) groups. Associations between SIG and clinicopathological variables, chemotherapy compliance, and overall survival (OS) were evaluated using ROC analysis, Kaplan-Meier curves, and Cox regression. Subgroup analyses stratified by tumor location (colon vs. rectum) were performed to assess prognostic heterogeneity.

**Results:**

Higher SIG scores correlated with elevated CEA (P=0.002), advanced TNM stage (P=0.001), and reduced chemotherapy compliance (64.0% non-compliant patients had SIG≥2, P<0.001). Multivariate analysis identified SIG (HR=2.24, P<0.001), CEA, tumor differentiation, and TNM stage as independent prognostic factors. SIG demonstrated superior prognostic accuracy (AUC=0.785) compared to NLR (0.713), mGPS (0.673), and TNM staging (0.675). Kaplan-Meier analysis revealed significant survival differences across SIG groups (5-year OS: 90.9% vs. 76.4% vs. 37.0%, P<0.001) and additional stratification within TNM stages. Subgroup analysis showed consistent prognostic efficacy of SIG in both colon and rectal cancers, with no significant interaction between SIG and tumor location (P=0.309).

**Conclusions:**

SIG outperforms existing biomarkers and complements TNM staging by capturing systemic inflammation-driven risk heterogeneity. Its prognostic consistency across colon and rectal cancers supports its utility as a universal tool for postoperative risk stratification, guiding personalized adjuvant therapy and surveillance strategies.

## 1 Introduction

Colorectal cancer (CRC) is one of the leading malignant tumors worldwide in terms of incidence and mortality rates (1). Although surgical resection remains the main curative treatment for colorectal cancer, the prognosis after surgery varies significantly among patients (2). Therefore, identifying biomarkers that can accurately predict long-term postoperative prognosis is crucial for developing personalized treatment strategies and follow-up plans.

In recent years, numerous studies have shown that systemic inflammation is closely associated with the occurrence, development, and prognosis of various solid tumors (3–5). Inflammatory responses play a complex role in the tumor microenvironment, promoting tumor growth and metastasis while also activating anti-tumor immune responses (6, 7). The degree of systemic inflammation is considered an important factor affecting the prognosis of colorectal cancer patients (8).

Currently, several indicators reflecting systemic inflammation have been used to evaluate the prognosis of colorectal cancer patients. Among them, the Neutrophil-to-lymphocyte ratio (NLR), a simple and easily obtainable indicator, has been widely used to predict the prognosis of colorectal cancer patients (9, 10). The NLR reflects the body’s inflammatory status and immune function, with higher NLR values often associated with poorer prognosis. On the other hand, the modified Glasgow Prognostic Score (mGPS) comprehensively assesses the patient’s inflammatory status and nutritional condition, also providing good prognostic value (11).

However, a single indicator may not fully reflect the patient’s systemic inflammatory status. Therefore, researchers have started combining multiple indicators to improve the accuracy of prognostic predictions. The systemic inflammation grade (SIG) is a newly proposed scoring system that combines NLR and mGPS (12). Preliminary studies have shown that SIG has good prognostic value in colon cancer patients, but its application value in colorectal cancer patients needs further validation.

Based on the above background, this study aims to retrospectively analyze the clinical data of colorectal cancer patients who underwent radical surgery to systematically evaluate the predictive effectiveness of SIG on long-term overall survival (OS). We will compare the prognostic value of SIG with that of NLR and mGPS, explore the relationship between SIG and clinicopathological characteristics, and analyze the predictive performance of SIG in different patient subgroups. Through this study, we hope to provide clinicians with a more comprehensive and accurate prognostic assessment tool to help formulate personalized postoperative follow-up and adjuvant treatment strategies, ultimately improving the long-term survival outcomes of colorectal cancer patients.

## 2 Methods

### 2.1 Study population

This retrospective study included 263 patients who underwent curative surgery for CRC at our hospital between January 2015 and December 2019.

Inclusion criteria:(1) Histologically confirmed colorectal cancer; (2) R0 resection with ≥12 lymph nodes examined; (3) TNM stage I–III; (4) Complete preoperative biochemical data.

Exclusion criteria: (1) Received preoperative chemotherapy or radiotherapy; (2) Presence of bowel obstruction or perforation; (3) Active inflammatory diseases or history of IBD; (4) Incomplete clinical data; (5) Loss to follow-up.

### 2.2 Data collection

General clinical data, including age, gender, history of hypertension, diabetes, abdominal surgery, tumor size, pathological stage, and differentiation grade, were collected. Preoperative laboratory tests (including routine blood tests, liver and kidney function tests, and tumor markers) were performed within 7 days prior to surgery to ensure the relevance of the data to the patients’ immediate preoperative status. TNM staging was based on the 8th edition of the TNM classification system (13). Postoperative chemotherapy regimens followed the 2021 NCCN guidelines (14), which recommend adjuvant chemotherapy for high-risk stage II patients (T4, vascular or perineural invasion, or poor differentiation) and all stage III patients. Chemotherapy compliance was classified as follows: full compliance (completion of 100% of the planned cycles with dose adjustment ≤20%), partial compliance (completion of 80%–99% of cycles), and noncompliance (completion of <80% of cycles).

### 2.3 Assessment and grouping of systemic inflammation status

Based on preoperative blood results, each patient’s NLR and mGPS were calculated to retrospectively assess systemic inflammation. The NLR was calculated using the formula: NLR = Neutrophil count (×10^9^/L)/Lymphocyte count (×10^9^/L). According to previous studies, NLR was divided into three groups (15): normal (NLR < 3), moderate (3 ≤ NLR < 5), and elevated (NLR ≥ 5). The mGPS grouping criteria (16, 17) were: CRP < 10 mg/L scored 0, CRP > 10 mg/L scored 1, and CRP > 10 mg/L with hypoalbuminemia (< 35 g/L) scored 2. SIG was formed by combining NLR and mGPS (18), categorizing patients into 0–4 levels. SIG=0: mGPS (0) and NLR < 3; SIG=1: mGPS (0) and 3 ≤ NLR < 5, or mGPS (1) and NLR < 3; SIG=2: mGPS (0) and NLR > 5, or mGPS (2) and NLR < 3, or mGPS (1) and 3 ≤ NLR < 5; SIG=3: mGPS (1) and NLR > 5, or mGPS (2) and 3 ≤ NLR < 5; SIG=4: mGPS (2) and NLR > 5. Based on previous research and clinical practice (18, 19), we categorized SIG scores into three groups: low (SIG = 0), medium (SIG = 1), and high (SIG ≥ 2). This grouping method better balances sample sizes and aligns with clinical application needs.

### 2.4 Follow-up and endpoint

A total of 302 eligible patients were initially enrolled in this study. Follow-up was conducted from 10 to 65 months (mean: 54.6 ± 10.81 months; median: 60 months), and continued until December 2019. Among these, 39 patients were lost to follow-up, resulting in complete data collection for 263 patients, with an attrition rate of 12.91%. Follow-up evaluations were scheduled at postoperative months 1, 3, 6, 9, and 12, and subsequently at 6-month intervals thereafter. Each follow-up visit included a physical examination, imaging studies, and laboratory tests. The primary endpoint was OS, defined as the time from surgery to death from any cause or the date of the last follow-up.

### 2.5 Statistical analysis

All statistical analyses were conducted using SPSS 26.0 (IBM Corp., Armonk, NY) and Sangerbox Tools (version 3.0, Sangerbox Biomedical Technology Co., China). Categorical variables were compared using the chi-square test or Fisher’s exact test, as appropriate. Survival outcomes were analyzed using the Kaplan-Meier method, and differences between groups were assessed by the log-rank test. Univariate and multivariate Cox proportional hazards models were employed to identify independent prognostic factors for OS, with variables showing P < 0.05 in univariate analysis included in the multivariate model. The prognostic performance of SIG, NLR, mGPS, and TNM staging was compared using ROC curves, with area under the curve (AUC) values and 95% confidence intervals (CIs) calculated and differences assessed via DeLong’s test. Subgroup analyses stratified by tumor location (colon vs. rectum) were performed, and interaction effects between SIG and tumor location were tested by incorporating an interaction term (SIG × tumor location) into the Cox regression model. All tests were two-tailed, and a P value < 0.05 was considered statistically significant.

## 3 Results

### 3.1 Patient demographics

This study included 263 postoperative CRC patients, with ages ranging from 27 to 88 years, and a median age of 66 years. Of the patients, 155 were male and 108 were female. Among them, 179 patients were alive, and 84 had died, resulting in a 5-year postoperative survival rate of 68.10%. The collected data were verified, with TNM staging distribution as follows: 64 patients in stage I, 105 in stage II, and 94 in stage III. Based on chemotherapy status, patients were divided into three groups: Group 0 (no chemotherapy required, n = 147); Group 1 (chemotherapy required and ≥80% of the planned chemotherapy cycles completed, n = 91); and Group 2 (chemotherapy required and <80% of the planned chemotherapy cycles completed, n = 25). **Table 1** provides a detailed summary of the clinical and pathological characteristics of the patients, including age, gender, tumor location, tumor size, and TNM stage.

**Table 1:** Comparison of clinical and pathological characteristics in CRC Patients with different SIG scores.

Variable	Total Count	SIG			χ^2^	P-value
		0 (n=99)	1 (n=72)	≥2 (n=92)		
Gender					1.589	0.452
Male	155	60	38	57		
Female	108	39	34	35		
Age (years)					2.468	0.291
≤66	136	57	33	46		
>66	127	42	39	46		
Diabetes					3.754	0.153
Yes	34	8	10	16		
No	229	91	62	76		
Hypertension					0.805	0.669
Yes	80	27	24	29		
No	183	72	48	63		
Tumor Location					1.36	0.510
Colon	175	68	50	57		
Rectum	88	31	22	35		
History of Abdominal Surgery					2.451	0.294
Yes	55	19	12	24		
No	208	80	60	68		
CEA					12.309	0.002^*^
<5ng/mL	194	81	57	56		
≥5ng/mL	69	18	15	36		
Tumor Diameter					1.576	0.455
<5cm	182	66	54	62		
≥5cm	81	33	18	30		
TNM Stage					18.947	0.001^*^
I	64	34	15	15		
II	105	45	25	35		
III	94	20	32	42		
Tumor Differentiation					5.552	0.062
High/Moderate	174	73	48	53		
Poor/Undifferentiated	89	26	24	39		
NLR					261.998	0.000^*^
<3	137	99	28	10		
3~5	62	0	44	18		
≥5	64	0	0	64		
mGPS					116.185	0.000^*^
0	180	99	44	37		
1	53	0	28	25		
2	30	0	0	30		
Chemotherapy					4.662	0.324
Group 0	147	63	38	46		
Group 1	91	30	26	35		
Group 2	25	6	8	11		

The symbol of * represents difference, indicating P < 0.05.

### 3.2 Association between SIG grouping and clinical pathological characteristics

A total of 263 CRC patients were divided into three groups based on their SIG scores: SIG = 0 (n = 99), SIG = 1 (n = 72), and SIG ≥ 2 (n = 92). A statistical analysis was performed to compare clinical characteristics, including gender, age, hypertension, diabetes, tumor location, tumor size, TNM stage, tumor differentiation, CEA levels, and other relevant factors across the low, medium, and high SIG groups. The results revealed significant differences between the groups in terms of CEA (χ² = 12.309, P = 0.002), TNM stage (χ² = 18.947, P = 0.001), NLR grouping (χ² = 261.998, P < 0.001), and mGPS (χ² = 116.185, P < 0.001). However, no significant differences were found between the groups regarding gender, age, hypertension, diabetes, tumor location, abdominal surgery history, tumor size, tumor differentiation, or chemotherapy status (P > 0.05). A detailed comparison of these results is presented in **Table 1**.

### 3.3 Univariate and multivariate cox regression analysis for OS in postoperative CRC patients

**Figure 1:**
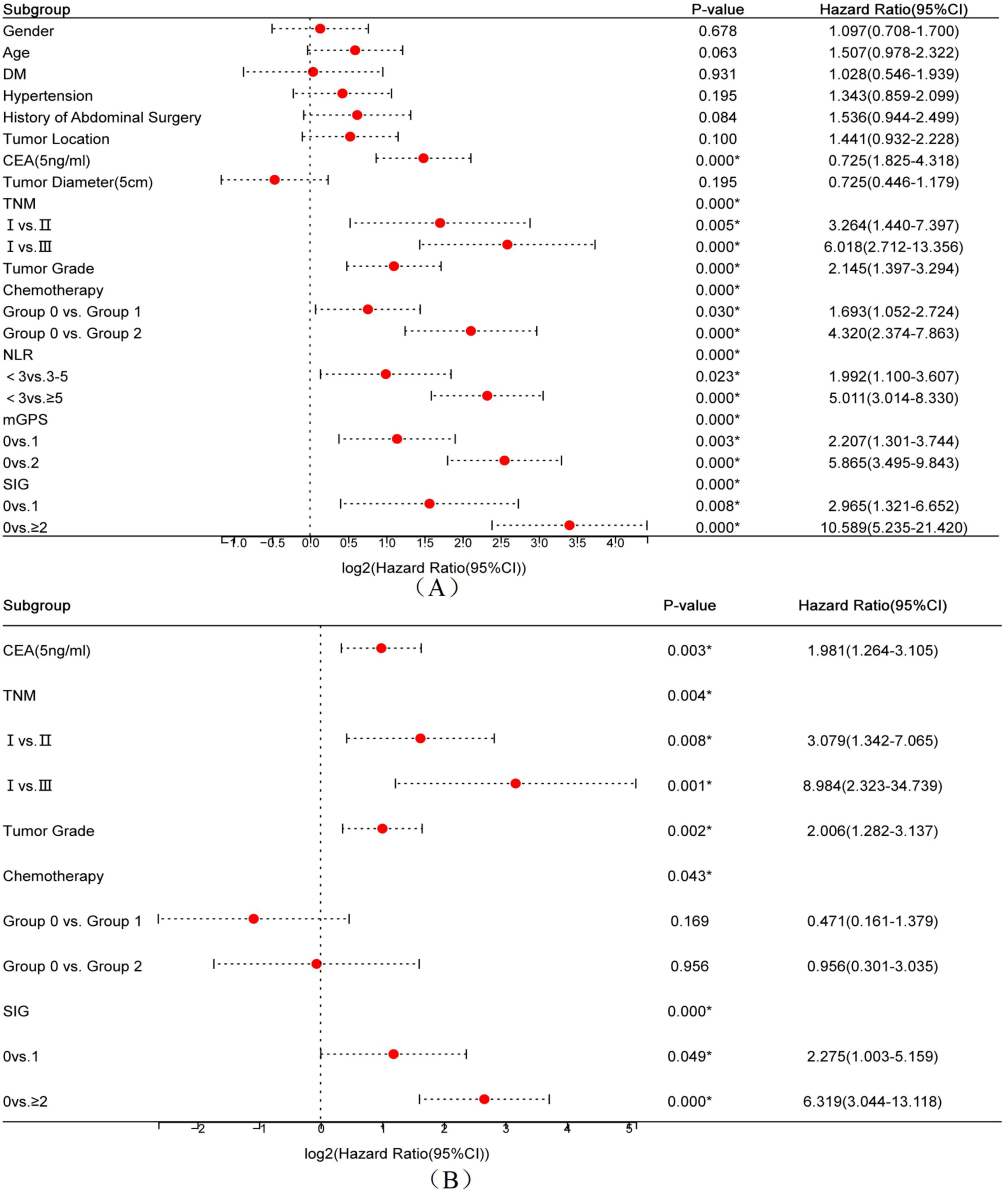
Forest plots of univariate **(A)** and multivariate **(B)** Cox regression analyses for overall survival in postoperative colorectal cancer patients.

Univariate Cox regression analysis was conducted to identify factors associated with OS in postoperative CRC patients. As shown in **Figure 1A**, the analysis revealed that CEA level, tumor size, TNM stage, chemotherapy status, tumor differentiation, NLR grouping, mGPS score, and SIG score were significantly associated with OS (all P < 0.05). Due to their inclusion in the composite SIG score, mGPS and NLR were not entered into the multivariate analysis. As shown in **Figure 1B**, the multivariate analysis identified CEA, TNM stage, tumor differentiation, and SIG as independent prognostic factors for OS in CRC patients (all P < 0.05). Specifically, lower CEA levels (<5 ng/mL), early TNM stage (stage I), high and moderately differentiated tumors, and a SIG score of 0 were associated with improved postoperative OS.

### 3.4 Association between chemotherapy compliance and preoperative SIG score

In the multivariate Cox regression analysis, although postoperative chemotherapy showed overall significance (P = 0.043), pairwise comparisons among subgroups did not reach statistical significance, and thus chemotherapy was not identified as an independent prognostic factor. To further evaluate the impact of preoperative inflammatory status on chemotherapy behavior, the association between preoperative SIG score and postoperative chemotherapy compliance was analyzed in 116 patients with indications for adjuvant chemotherapy (excluding those not requiring chemotherapy). As shown in **Table 2**, a significant association was observed between chemotherapy compliance and preoperative SIG score (χ² = 32.68, P < 0.001). Among patients with full compliance (n = 66), 66.7% had a SIG score of 0 (n = 44), and only 9.1% had a score ≥2 (n = 6). In contrast, among patients with non-compliance (n = 25), the proportion of those with SIG ≥2 increased markedly to 64.0% (n = 16). These results suggest that elevated preoperative SIG scores may be associated with reduced compliance to postoperative chemotherapy.

**Table 2:** Distribution of preoperative SIG scores according to chemotherapy compliance.

Chemotherapy Compliance	Total Count	SIG			χ²	P-value
		0 (n=58)	1 (n=29)	≥2 (n=29)	32.68	<0.001
Full compliance	66	44	16	6		
Partial compliance	25	10	8	7		
Non-compliance	25	4	5	16		

### 3.5 Predictive Ability of SIG for OS in CRC Patients

**Figure 2:**
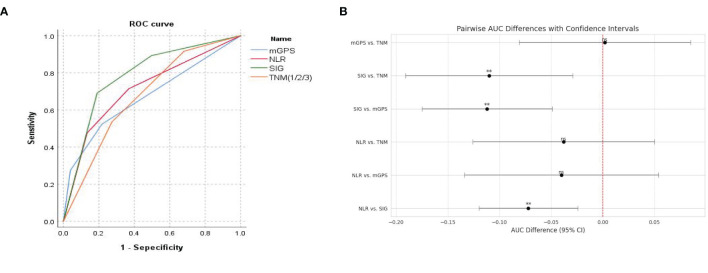
**(A)** ROC curve for predicting postoperative survival in CRC patients; **(B)** Comparison of AUC values for predicting postoperative survival in CRC patients.

ROC curves for preoperative NLR, mGPS, SIG, and TNM stage were constructed with postoperative survival status as the outcome variable (**Figure 2A**). The results showed that the AUC for NLR was 0.713 (95% CI: 0.648-0.777), for mGPS was 0.673 (95% CI: 0.609-0.736), for SIG was 0.785 (95% CI: 0.729-0.840), and for TNM was 0.675 (95% CI: 0.612-0.737). Statistical comparisons of the AUC values (**Figure 2B**) revealed no significant differences between NLR and mGPS (P = 0.404), between NLR and TNM (P = 0.395), or between mGPS and TNM (P = 0.960). However, significant differences were observed between SIG and NLR, mGPS, and TNM (P = 0.004, P = 0.001, P = 0.008, respectively).

### 3.6 Association of SIG with postoperative OS in CRC patients: Kaplan-Meier survival analysis Kaplan-Meier

**Figure 3:**
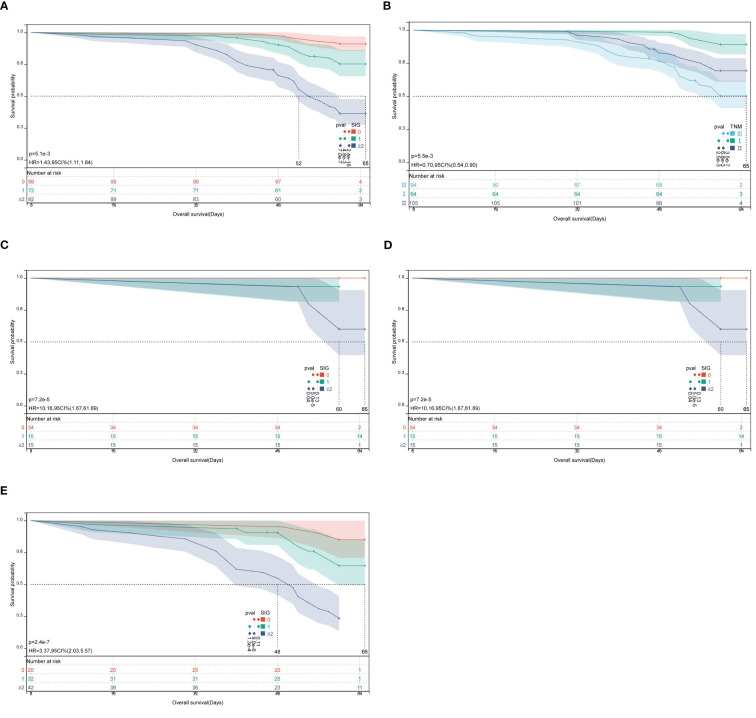
Kaplan-Meier survival curves for OS in CRC patients according to SIG score groups and TNM stage. **(A)** Survival analysis of low, middle, and high SIG score groups; **(B)** Survival analysis based on TNM stage (I, II, III); **(C–E)** Survival analysis of different SIG score groups within TNM stages I, II, and III.

Kaplan-Meier survival curves revealed significant differences in 5-year OS among patients with low, medium, and high SIG scores (**Figure 3A**). In the low SIG group (n = 99), 90 patients survived (survival rate: 90.9%); in the medium SIG group (n = 72), 55 patients survived (survival rate: 76.4%); and in the high SIG group (≥2, n = 92), only 34 patients survived (survival rate: 37.0%). The survival rates in the low and medium SIG groups were significantly higher than those in the high SIG group (χ^2^ = 77.875, P < 0.001). In addition, Kaplan-Meier survival analysis based on TNM stage also showed significant differences in OS among stages I, II, and III (**Figure 3B**). In stage I, 64 patients were included, with 7 deaths, yielding a survival rate of 89.1%; in stage II, 105 patients were included, with 32 deaths, yielding a survival rate of 69.5%; and in stage III, 94 patients were included, with 45 deaths, yielding a survival rate of 52.1%. The survival rate differences between TNM stages were statistically significant (χ^2^ = 26.884, P < 0.001). Finally, within TNM stages I (**Figure 3C**), II (**Figure 3D**), and III (**Figure 3E**), Kaplan-Meier survival analysis demonstrated significant differences in 5-year OS across different SIG score groups. For stage I (χ^2^ = 17.880, P < 0.001), stage II (χ^2^ = 21.583, P < 0.001), and stage III (χ^2^ = 30.465, P < 0.001), survival rates varied significantly by SIG score groups.

### 3.7 Prognostic heterogeneity of SIG in colon cancer vs. rectal cancer

**Figure 4:**
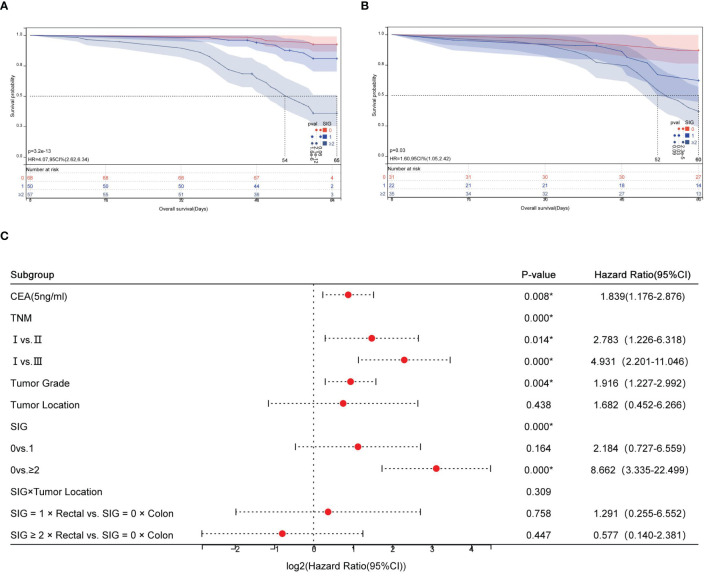
Prognostic stratification of overall survival by SIG and tumor location in CRC. **(A)** Kaplan-Meier survival curves for colon cancer patients stratified into low, medium, and high SIG score groups; **(B)** Kaplan-Meier survival curves for rectal cancer patients stratified into low, medium, and high SIG score groups; **(C)** Forest plot of interaction effects between SIG and tumor location.

Kaplan-Meier survival analysis revealed distinct survival outcomes stratified by SIG scores in both colon and rectal cancer subgroups. For colon cancer patients (n=175), the 5-year survival rates significantly declined with increasing SIG scores: 92.6% (SIG=0), 82.0% (SIG=1), and 36.8% (SIG≥2) (χ^2^ = 62.705, P<0.001). Median survival time was not reached for SIG=0 and SIG=1 groups (indicating >50% of patients survived beyond the study period), while SIG≥2 patients exhibited a median survival of 56.0 months (95% CI: 49.8–62.2) (**Figure 4A**). Similarly, in rectal cancer (n=88), survival rates decreased from 87.1% (SIG=0) to 37.1% (SIG≥2) (χ^2^ = 17.798, P<0.001), with SIG≥2 patients showing a median survival of 54.0 months (95% CI: 48.2–59.8) (**Figure 4B**).

Cox regression analysis demonstrated that high SIG scores (SIG≥2) independently predicted poorer survival in colon cancer (HR=8.66, 95% CI: 3.34–22.50, P<0.001). Interaction analysis revealed no statistically significant differential prognostic effect of SIG between colon and rectal cancers (SIG×Tumor Location interaction χ^2^ = 2.350, P=0.309; **Figure 4C**).

## 4 Discussion

This study evaluated the prognostic value of the SIG in predicting long-term survival among 263 patients with CRC who underwent curative resection. The findings demonstrated that SIG, a composite score integrating the NLR and the mGPS, outperformed individual inflammatory markers and conventional TNM staging in prognostic capability. Notably, higher SIG scores were significantly associated with elevated CEA levels (P = 0.002) and advanced TNM stages (P = 0.001). As one of the most commonly used tumor markers in CRC, elevated CEA is generally linked to disease progression and poor outcomes (20). The correlation between SIG and CEA further underscores the prognostic potential of SIG. Moreover, the association between SIG and TNM staging suggests that systemic inflammation tends to worsen with tumor progression, aligning with previous reports indicating more severe inflammation in patients with advanced cancer (21, 22). These observations aslo support the hypothesis that inflammation-driven pathways may enhance CRC aggressiveness, as reflected in the link between higher SIG scores and adverse clinicopathological features.

Univariate Cox analysis identified NLR, mGPS, and SIG as significant predictors of OS. Multivariate Cox regression analysis revealed that CEA level, tumor differentiation, TNM stage, and SIG score were independent prognostic factors for postoperative OS in CRC patients. These results highlight the synergistic prognostic contribution of systemic inflammation, represented by SIG, and tumor biology, including differentiation status and TNM stage. Importantly, although NLR and mGPS were significant predictors in the univariate analysis, they were excluded from the multivariate model due to their collinearity with SIG, which integrates both variables. This approach highlights the advantage of SIG as a composite index that encapsulates both systemic inflammation and nutritional status, offering a more comprehensive reflection of the host–tumor interaction while minimizing model redundancy and improving clinical interpretability (23). As shown in **Figure 2**, with an AUC of 0.785, SIG showed enhanced predictive ability relative to NLR, mGPS, and TNM staging, supporting its role as a complementary prognostic biomarker.

A notable finding was the inverse relationship between preoperative SIG scores and chemotherapy compliance (P < 0.001), with 64.0% of non-compliant patients classified as SIG ≥ 2. This suggests that systemic inflammation may compromise treatment tolerance, potentially due to frailty, immune dysfunction, or exacerbated chemotherapy-related toxicity (24). These results highlight the need for preoperative anti-inflammatory interventions, such as nutritional support or immunomodulatory therapies, to enhance treatment compliance and efficacy. Additionally, SIG was not significantly associated with demographic factors such as age, sex, or hypertension, indicating its broad applicability across diverse patient populations and independence from common clinical variables.

While TNM staging remains essential, SIG adds prognostic refinement by identifying high-risk patients within the same TNM stages. Kaplan–Meier survival analyses further emphasized the additive prognostic value of SIG alongside TNM staging. While TNM staging effectively stratified patients by anatomical tumor burden (e.g., 5-year OS: 89.1% for stage I vs. 52.1% for stage III, P < 0.001), SIG provided additional prognostic granularity within each TNM subgroup. For instance, among stage III patients, those with SIG ≥ 2 had a markedly lower 5-year OS (23.8%) than those with lower SIG scores, indicating that systemic inflammation exacerbates prognosis even in advanced stages. Conversely, early-stage (TNM I) patients with high SIG scores (≥ 2) exhibited a 5-year OS of 60.0%, comparable to that of some late-stage (TNM III) patients, suggesting that inflammation-driven biological aggressiveness may transcend anatomical staging in determining prognosis (25). While TNM staging reflects tumor burden, SIG captures dynamic host response—this dual-axis model has gained increasing support in recent literature (18, 19, 26). For example, Golder et al (18). demonstrated that SIG stratified survival within TNM-defined groups in a large cohor; our findings extend this evidence to a Chinese population, confirming the cross-ethnic relevance of SIG. Integrating both systems may help clinicians better identify high-risk patients who could benefit from intensified adjuvant therapy or monitoring, even within conventionally “favorable” TNM categories, thereby addressing the heterogeneity masked by anatomical staging alone.

Despite significant differences in anatomical features (e.g., primary location), molecular profiles (e.g., microsatellite instability [MSI] in proximal colon vs. chromosomal instability [CIN] in distal colon/rectum), and therapeutic approaches (e.g., neoadjuvant chemoradiotherapy for rectal cancer) between colon and rectal cancers (27–29), our interaction analysis (SIG × tumor location, P = 0.309) demonstrated consistent prognostic efficacy of SIG across both subsites. This finding suggests that systemic inflammatory responses, as comprehensively evaluated by SIG, may act as a common mechanism transcending subsite boundaries to drive CRC progression. Biologically, chronic inflammation promotes tumor immune evasion and metastatic dissemination through synergistic effects, including activation of immunosuppressive pathways (e.g., PD-1/PD-L1, TGF-β), release of pro-inflammatory cytokines (e.g., IL-6, TNF-α), and dysregulation of nutritional metabolism (30, 31), thereby underpinning SIG’s stable predictive value across anatomical subsites. Consequently, SIG not only complements traditional anatomical stratification but also serves as a universal tool for preoperative risk assessment. Future studies should integrate SIG with molecular subtypes (e.g., MSI/CIN status), immune microenvironment features (e.g., CD8+ T-cell infiltration), and gut microbiome biomarkers to construct multidimensional prognostic models, ultimately optimizing individualized therapeutic strategies.

For future CRC management, patients with high SIG scores, especially those in stage III, may benefit from more aggressive adjuvant chemotherapy to reduce the risk of residual disease and recurrence. More intensive follow-up protocols, including imaging and tumor marker assessments every three months, may also be warranted to facilitate early detection of recurrence or metastasis. Furthermore, the elevated systemic inflammation represented by high SIG scores suggests that enhanced preoperative measures, such as immunomodulatory interventions and nutritional support, could help mitigate inflammation and potentially improve overall outcomes. Future research should aim to validate the prognostic value of SIG for postoperative OS and disease-free survival (DFS) in large-scale, multicenter studies and explore the combined utility of SIG with other prognostic indicators. This study has several limitations. First, it primarily assessed the prognostic value of NLR, mGPS, and SIG, without including other inflammatory markers. Second, the single-center retrospective design may introduce selection bias. Lastly, although the three-tiered SIG classification demonstrated strong prognostic performance in this study, the optimal stratification method requires further validation in larger cohorts. Addressing these limitations will be a key focus of our future research, aimed at developing more comprehensive and individualized treatment strategies.

## 5 Conclusion

This study demonstrates that the SIG, which integrates the NLR and mGPS, serves as a robust prognostic biomarker for CRC, with superior predictive accuracy (AUC = 0.785) compared to individual inflammatory markers and TNM staging. Despite inherent anatomical and molecular heterogeneity between colon and rectal cancers, SIG exhibited consistent prognostic efficacy across both subsites (interaction P = 0.309), and high SIG scores independently predicted mortality risk in CRC patients. Elevated SIG scores were significantly associated with reduced chemotherapy compliance (64.0% non-compliance in patients with SIG ≥ 2) and adverse clinicopathological features, underscoring its clinical utility in identifying high-risk populations for intensified adjuvant therapy or preoperative anti-inflammatory interventions. Future multicenter studies are warranted to validate the generalizability of SIG and explore its integration with molecular subtypes (e.g., consensus molecular subtypes, CMS) and gut microbiota dynamics to refine precision oncology strategies.

## Data Availability

The data analyzed in this study is subject to the following licenses/restrictions: The data that support the findings of this study are available from the corresponding author upon reasonable request. Requests to access these datasets should be directed to Honggang Wang honggangtz@163.com.
